# Supplementary material for: Mites Living in the Nests of the White Stork and Black Stork in Microhabitats of the Forest Environment and Agrocenoses
Source: Animals (Basel). 2023 Oct 12;13(20):3189. doi: 10.3390/ani13203189 (PMC10603721; doi:10.3390/ani13203189)
Supplement: Supplementary file 1 [file animals-13-03189-s001.zip › animals-2621181-supplementary.pdf]

animals

MDPI

Supplementary Materials

Table S1. List of Oribatida taxons and their preferences, found in the nests of the White Stork and the nests of the Black Stork.

<table>
<tr><th colspan="2">Taxon</th><th rowspan="2">Habitat Preferences [95,96,99]</th><th rowspan="2">Dietary preferences [95,96]</th><th rowspan="2">Prevalence [95,97,101,102]</th><th rowspan="2">Reproduction [98-100]</th></tr>
<tr><th>White Stork</th><th>Black Stork</th></tr>
<tr><td colspan="2">Scheloribates laevigatus (C. L. Koch, 1835)</td><td>Eur [1], Mea</td><td>Pan [2], Cop</td><td>Hol [3]</td><td>S [4]</td></tr>
<tr><td colspan="2">Ramusella fasciata (Paoli, 1908)</td><td>nd. [5]</td><td>nd.</td><td>Hol</td><td>nd.</td></tr>
<tr><td colspan="2">Punctoribates punctum (C.L. Koch, 1839)</td><td>Eur, Wood, Arb, Mos, Gras, Mes</td><td>Pan</td><td>Cosm</td><td>S</td></tr>
<tr><td colspan="2">Tectocepheus velatus (Michael, 1880)</td><td>Eur</td><td>Mic</td><td>Cosm</td><td>P</td></tr>
<tr><td colspan="2">Oribatula exilis (Nicolet, 1855)</td><td>Eur, Arb, Mos, Xer</td><td>Mic</td><td>Hol</td><td>S</td></tr>
<tr><td colspan="2">Liebstadia similis (Michael 1888)</td><td>Wood, Gras, Hyg</td><td>Pan</td><td>Hol</td><td>nd.</td></tr>
<tr><td colspan="2">Oppia denticulata (Canestrini, 1882)</td><td>Eur</td><td>nd.</td><td>Pal</td><td>nd.</td></tr>
<tr><td>Oribatula pannonica (Willmann, 1949)</td><td></td><td>Xer, Mos, Hal</td><td>Mic</td><td>Euro</td><td>S</td></tr>
<tr><td colspan="2">Trichoribates trimaculatus (C. L. Koch, 1835)</td><td>Arb, Mos, Gras, Xer</td><td>Pan</td><td>Hol</td><td>nd.</td></tr>
<tr><td colspan="2">Eupelops occultus (C. L. Koch, 1835)</td><td>Gras, Tyr</td><td>Pan</td><td>Pal</td><td>S</td></tr>
<tr><td colspan="2">Galumna obvia (Berlese, 1915)</td><td>Gras</td><td>Pan</td><td>Hol</td><td>S</td></tr>
<tr><td colspan="2">Achipteria nitens (Nicolet, 1855)</td><td>Wood, Mpl</td><td>Pan</td><td>Pal</td><td>S</td></tr>
<tr><td colspan="2">Achipteria coleoptrata (Linné, 1758)</td><td>Eur, Wood</td><td>Mac, Pan</td><td>Hol</td><td>S</td></tr>
<tr><td colspan="2">Platynothrus peltifer (C.L. Koch, 1839)</td><td>Eur, Wood, Arb, Pem, Hyg, Gras,</td><td>Pan</td><td>Cosm</td><td>P</td></tr>
<tr><td>Scheloribates pallidulus (C.L. Koch, 1841)</td><td></td><td>Eur, Wood, Gras, Pem, Hyg</td><td>Pan</td><td>Cosm</td><td>S</td></tr>
<tr><td>Tectoribates ornatus (Schuster, 1958)</td><td></td><td>Xer, Gras, Hal</td><td>Mic</td><td>Pal</td><td>nd.</td></tr>
<tr><td>Trichoribates incisellus (Kramer, 1897)</td><td></td><td>Gras, Hal</td><td>Pan</td><td>Pal</td><td>nd.</td></tr>
<tr><td colspan="2">Pergalumna nervosa (Berlese, 1914)</td><td>Eur, Wood, Hyg, Gras, Pem, Xer</td><td>Pan</td><td>Cosm</td><td>S</td></tr>
<tr><td colspan="2">Chamobates cuspidatus (Michael, 1884)</td><td>Wood, Tyr</td><td>Pan</td><td>Hol</td><td>nd.</td></tr>
<tr><td>Neoribates aurantiacus (Oudemans, 1914)</td><td></td><td>Wood, Gras</td><td>nd.</td><td>Hol</td><td>nd.</td></tr>
<tr><td>Diapterobates humeralis (Hermann, 1804)</td><td></td><td>Wood, Arb, Gras, Xer</td><td>Mic</td><td>Hol</td><td>S</td></tr>
<tr><td>Eupelops subuliger (Berlese, 1916)</td><td></td><td>Wood, Hig</td><td>nd.</td><td>Euro</td><td>S</td></tr>
<tr><td colspan="2">Oppiella nova (Oudemans, 1902)</td><td>Eur</td><td>Mic</td><td>Cosm</td><td>P</td></tr>
</table>

Eniochtchonius minutissimus (Berlese, 1903)		Eur, Wood	Mic	Cosm	P
Ceratozetes gracillis (Michael, 1884)		Wood, Moo	Pan	Hol	nd.
Carabodes labyrinthicus (Michael, 1879)		Eur, Wood, Arb, Mos	Mac	Hol	S
Liacarus coracinus (C.L. Koch, 1841)		Wood, Gras	Pan	Pal	nd.
Spatiodamaeus verticilipes (Nicolet, 1855)		Wood, Mos	nd.	Hol	nd.
Eupelops plicatus (C.L. Koch, 1836)		Wood, Arb	nd.	Hol	S
Nothrus silvestris (Nicolet, 1855)		Eur, Wood	Mac, Pan	Cosm	P
Minutozetes pseudofusiger (Schweizer, 1922)		Arb, Mos, Xer	nd.	Pal	nd.
Phthiracarus sp. (Perty, 1841)		-	-	-	-
Punctoribates hexagonus (Berlese, 1908)		Gras, Hal, Tyr	nd.	Hol	nd.
Ramusella 2ectina (Willmann, 1928)		Pem, Wood	nd.	Euro	nd.
Peloptulus phaenotus (C. L. Koch, 1844)		Gras, Hal, Ree	nd.	Pal	nd.
Nanhermannia nana (Nicolet, 1855)		Wood, Gras, Peb, Hyg	Mic, Pan	Cosm	P
Adoristes ovatus (C.L. Koch, 1839)		Eur, Wood, Pem	Pan	Hol	S
	Ramusella clavipectinata (Michael, 1885)	Hyg, Gras	Pan	Hol	nd.
	Oppiella subpectinata (Oudemans, 1900)	Eur, Wood,	Mic	Hol	S
	Acrogalumna longipluma (Berlese, 1904)	Eur, Wood,	Pan	Cosm	nd.
	Oribella 2ectinate (Michael, 1885)	Cav, Mne	Pan	Euro	nd.
	Suctobelbella subtrigona (Oudemans, 1916)	Eur, Wood	Pan	Cosm	P
	Suctobelbella sarekensis (Forsslund, 1941)	Eur, Wood, Gras, Rud, Hyg	Pan	Hol	P
	Autogneta longilamellata (Michael, 1885)	Wood	Mic	Hol	S

	Phthiracarus italicus (Oudemans, 1906)	Hyg, Wood, Gras	Mac	Euro	S
	Scheloribates initialis (Berlese, 1908)	Eur	Mic, Pan	Cosm	S
	Hypochthonius rufulus (C.L. Koch, 1835)	Wood, Gras, Hyg, Pem	Mic, Pan, Nec	Hol	P
	Liebstadia humerata (Sellnick, 1928)	Arb, Mos, Xer	Pan	Hol	S
	Steganacarus carinatus (C.L. Koch, 1841)	Wood, Xer, Ant	Mac	Pal	S
	Subiasella quadrimaculata (Evans, 1952)	Hum	nd.	Pal	nd.
	Phauloppia rauschenensis (Sellnick, 1908)	Arb	nd.	Euro	nd.
	Microppia minus (Paoli, 1908)	Eur, Arb, Xer	Pan	Cosm	P
	Carabodes willmani (Bernini, 1975)	Wood, Mos, Lic, Moor	Mac	Hol	S
	Licneremaeus licnophorus (Michael, 1882)	Wood, Arb, Mos, Xer	Mic	Pal	S
	Licnodamaeus pulcherrimus (Paoli, 1908)	Xer	Mac	Pal	nd.
	Eueremaeus oblongus (C.L. Koch, 1835)	Wood, Arb, Mos, Xer	Mic	Hol	S
	Oribatella reticulata (Berlese, 1916)	Wood, Arb, Mos	nd.	Hol	S
	Carabodes ornatus (Štorkán, 1925)	Wood, Hyg	Mac, Pan	Pal	S
	Furcoribula furcillata (Nordenskiöld, 1901)	Wood, Ant	Pan	Hol	S
	Metabelba pulverosa (Strenzke, 1953)	Eur, Wood, Mos, Pem, Mes	Mic	Hol	S
	Zetorchestes falzonii (Coggi, 1898)	Wood, Lic, Mos,	nd.	Euro	nd.
	Fuscozetes fuscipes (C. L. Koch, 1844)	Hyg, Gras, Wood, Tyr	Pan, Nec	Hol	nd.

Weigmann (2006) [95], Schatz (1983) [96], Bernini et al. (1987) [97], Domes-Wehner (2009) [98], Fischer et al. (2010, 2014) [99,100], Weigmann and Schatz (2015) [101], Schatz and Fischer (2016)

[102]; [1]Ant—ant nests; Arb—arboreal; Cav—caves; Eur—eurytopic; Gras—grassland; Hal—halophilic; Hig—highland; Hum—humus-rich soils and compost; Hyg—hygrophilous; Lic—lichens; Mea—meadow; Mes—mesohygrophilous; Mne—mouse nests; Moor—moorland; Mos—mosses; Mpl—mesophilic plant litter; Peb—peat-bog; Pem—peat mosses; Ree—reeds; Rud—ruderal; Tyr—tyrphobiont; Wood—woodland; Xer—xerophilic; [2]Cop—coprophagous; Mac—macrophytophagou; Mic—microphytophagous; Nec—necrophagous; Pan—panphytophagous; [3] Cosm—cosmopolitan; Euro—European; Hol—Holarctic; Pal—Palearctic; [4] P—parthenogenetic, S—sexual; [5] nd.—no data.
